# Targeted manipulation of the sortilin–progranulin axis rescues progranulin haploinsufficiency

**DOI:** 10.1093/hmg/ddt534

**Published:** 2013-10-26

**Authors:** Wing C. Lee, Sandra Almeida, Mercedes Prudencio, Thomas R. Caulfield, Yong-Jie Zhang, William M. Tay, Peter O. Bauer, Jeannie Chew, Hiroki Sasaguri, Karen R. Jansen-West, Tania F. Gendron, Caroline T. Stetler, NiCole Finch, Ian R. Mackenzie, Rosa Rademakers, Fen-Biao Gao, Leonard Petrucelli

**Affiliations:** 1 Department of Neuroscience, Mayo Clinic Jacksonville, 4500 San Pablo Rd S, Jacksonville, FL 32224, USA,; 2 Department of Neurology, University of Massachusetts Medical School, Worcester, MA 01605, USA; 3 Department of Pathology, University of British Columbia, 2211 Wesbrook Mall, Vancouver V6T 2B5, British Columbia, Canada

## Abstract

Progranulin (*GRN*) mutations causing haploinsufficiency are a major cause of frontotemporal lobar degeneration (FTLD-TDP). Recent discoveries demonstrating sortilin (SORT1) is a neuronal receptor for PGRN endocytosis and a determinant of plasma PGRN levels portend the development of enhancers targeting the SORT1–PGRN axis. We demonstrate the preclinical efficacy of several approaches through which impairing PGRN's interaction with SORT1 restores extracellular PGRN levels. Our report is the first to demonstrate the efficacy of enhancing PGRN levels in iPSC neurons derived from frontotemporal dementia (FTD) patients with PGRN deficiency. We validate a small molecule preferentially increases extracellular PGRN by reducing SORT1 levels in various mammalian cell lines and patient-derived iPSC neurons and lymphocytes. We further demonstrate that SORT1 antagonists and a small-molecule binder of PGRN_588–593_, residues critical for PGRN–SORT1 binding, inhibit SORT1-mediated PGRN endocytosis. Collectively, our data demonstrate that the SORT1–PGRN axis is a viable target for PGRN-based therapy, particularly in FTD-*GRN* patients.

## INTRODUCTION

Progranulin (PGRN), a multi-functional secreted glycoprotein, plays key roles in various biological processes (1,2) and, when deficient, leads to frontotemporal dementia (FTD). Individuals carrying a null or loss-of-function allele in *GRN*, the gene encoding PGRN, suffer from PGRN haploinsufficiency, which is a major cause of the most common pathological subtype of FTD, frontotemporal lobar degeneration (FTLD-TDP) (3,4). While the mechanisms linking loss of PGRN function and disease pathogenesis remain unclear, evidence from molecular and cellular studies suggests that decreased levels of extracellular PGRN (exPGRN) are relevant to disease pathogenesis. For example, supplementation of exogenous PGRN in culture medium rescues neurite outgrowth deficits observed in *Grn*^−/−^ neuronal cultures (5), facilitates wound healing by promoting the accumulation of neutrophils, macrophages and fibroblasts (6) and inhibits neutrophilic inflammation *in vivo* (7). In addition, the 69 pathogenic loss-of-function mutations in *GRN* reported so far account for ∼4–26% of familial FTD cases and 1–12% of sporadic cases worldwide (4,8–15). Several disease-related missense mutations have also been identified and appear to be associated with reduced PGRN secretion (16). As such, drug discovery efforts aimed at enhancing PGRN levels in patients with FTD with *GRN* mutations (FTD-*GRN)* is of great interest to the scientific community (17,18). Exciting new research by our group and others demonstrates the interaction between PGRN and sortilin (SORT1), a neuronal receptor that mediates extracellular PGRN clearance via an endocytosis mechanism (19), is a promising target. For example, while SORT1 is an important regulator of PGRN levels (20), PGRN's neurotrophic and neuroprotective effects are SORT1 independent (5,21), providing assurance that the PGRN–SORT1 axis is a viable target for drug discovery efforts aimed at identifying exPGRN enhancers.

Herein, we identify and validate several therapeutic strategies—the development of SORT1 expression suppressors, SORT1 antagonists and small-molecule PGRN-specific binders—to reduce SORT1-mediated endocytosis, thereby enhancing exPGRN levels in relevant disease models.

## RESULTS

### Pharmacological suppression of SORT1 expression increases extracellular PGRN in mammalian cell lines

**Figure 1.**
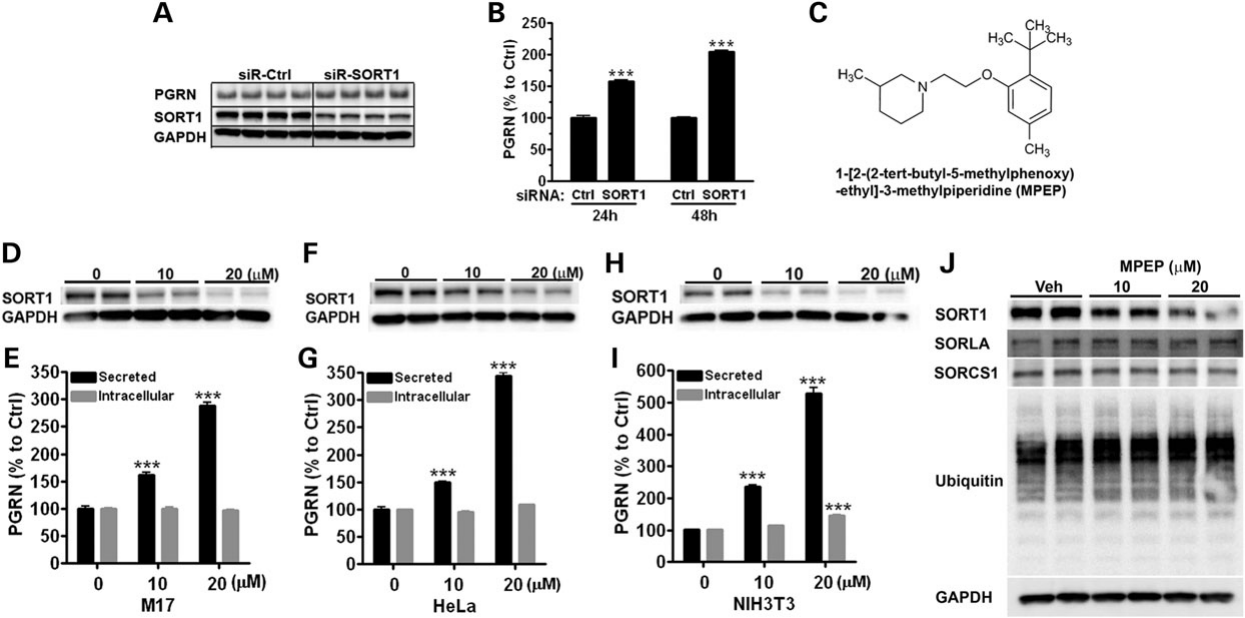
MPEP decreases SORT1 expression and increases extracellular PGRN in mammalian cell lines. (**A** and **B**) M17 cells were treated with control siRNA (siR-Ctrl) or gene-specific *SORT1* siRNA (siR-SORT1). (A) Intracellular levels of PGRN, SORT1 and GAPDH were evaluated by western blot at a 48 h time-point. (B) Suppression of SORT1 levels increased extracellular PGRN levels. (**C**) Chemical name and structure of MPEP. (**D**–**I**) Treatment of M17 cells (D and E), HeLa cells (F and G) or NIH3T3 cells (H and I) with MPEP for 24 h dose dependently reduced SORT1 levels (D, F and H) and increased exPGRN levels (E, G and I) at 10 and 20 μM. (**J**) Under the same conditions, MPEP did not affect levels of SORLA, SORCS1 and ubiquitinated proteins in M17 cells. ***P < 0.001 versus vehicle control, analysis performed by one-way ANOVA followed by Tukey's post-test.

Recent genetic evidence implicating SORT1 as an important regulator of GRN levels in serum (20) and the finding that ablation of Sort1 in *Grn*^+/−^ mice restores Pgrn in brain to normal levels (19) support the notion that pharmacological suppression of SORT1 expression in the brain may be a potential therapeutic approach for upregulating PGRN levels. Prior to investigating the use of SORT1 protein suppression as a PGRN enhancer, we first confirmed that SORT1 downregulation enhances PGRN levels in culture. To this end, SORT1 expression was reduced by treating M17 cells with SORT1-specific silencing RNA (siRNA) (Fig. 1A), which resulted in a significant increase in exPGRN levels in a time-dependent manner (Fig. 1B). While previous reports suggested the major mechanism of such observed rescue effects was due to a reduction of SORT1-mediated PGRN endocytosis, additional mechanisms, such as induction of PGRN secretion, had not been ruled out. To address this gap in our knowledge, we also evaluated intracellular PGRN levels and detected no significant changes (Fig. 1A), which indicates the boost in exPGRN levels is likely due to inhibition of endocytosis.

To evaluate the capacity of small molecules to suppress SORT1 levels, we screened a commercial compound library and identified a bioactive compound, 1-[2-(2-tert-butyl-5-methylphenoxy)-ethyl]-3-methylpiperidine, termed MPEP (Fig. 1C), that dose dependently reduces SORT1 levels in a mammalian cell lines. MPEP treatment for 24 h reduced SORT1 (Fig. 1D) and increased exPGRN up to 3-fold at a 20 μM dose (Fig. 1E). A similar effect was observed in HeLa cells (Fig. 1F and G) and in NIH3T3 cells, a mouse fibroblast line (Fig. 1H and I). To determine the specificity of MPEP, we also examined the effect of MPEP on other sortilin-related proteins: sortilin-related LDLR class A repeats-containing receptor (SORLA) and sortilin-related VPS10 domain-containing receptor 1 (SORCS1) (Fig. 1J). In addition, we examined levels of ubiquitinated proteins following MPEP treatment to determine whether this drug influences proteasomal degradation of proteins (Fig. 1J). That MPEP did not significantly alter the levels of any of these targets except for SORT1 provides evidence of its specificity. Moreover, under the same conditions, MPEP neither increased *GRN* mRNA nor suppressed *SORT1* mRNA levels indicating the effect is transcription independent (Supplementary Material, Fig. S1A). Rather, the significant decrease in SORT1 protein expression as early as 2 h post-MPEP treatment suggests MPEP effectively suppresses SORT1 levels by increasing SORT1 degradation (Supplementary Material, Fig. S1C). Finally, treatment of M17 cells with high doses of rPGRN, which unlike MPEP does not reduce SORT1 expression, rules out the possibility that MPEP acts via autocrine regulation (Supplementary Material, Fig. S1D).

### Pharmacological response to the small-molecule MPEP rescues PGRN haploinsufficiency in iPSC-neurons and lymphoblastoid cells derived from FTD patients

To validate the effect of MPEP in an authentic neuronal model of the disease, we used recently developed induced pluripotent stem cells (iPSCs) derived from FTD patients with a novel heterozygous GRN mutation (22). Because iPSCs are derived directly from somatic tissues of patients, the differentiated neuronal cells represent a more physiologically relevant model of the disease and platform for testing therapeutics. Like many other neurodegenerative disease iPSCs models, including ALS (23), AD (24) and PD (25), the FTD-*GRN* iPSCs neurons display the expected molecular phenotype caused by the inherited mutation (i.e. PGRN haploinsufficiency). For example, iPSC lines derived from FTD patients with the PGRN S116X mutation were confirmed to have 50% lower *GRN* mRNA levels than in non-carrier controls. Differentiation of the iPSC lines into PGRN S116X neurons yielded *GRN* mRNA that are ∼41% lower than in control and sporadic FTD neurons (22).

**Figure 2.**
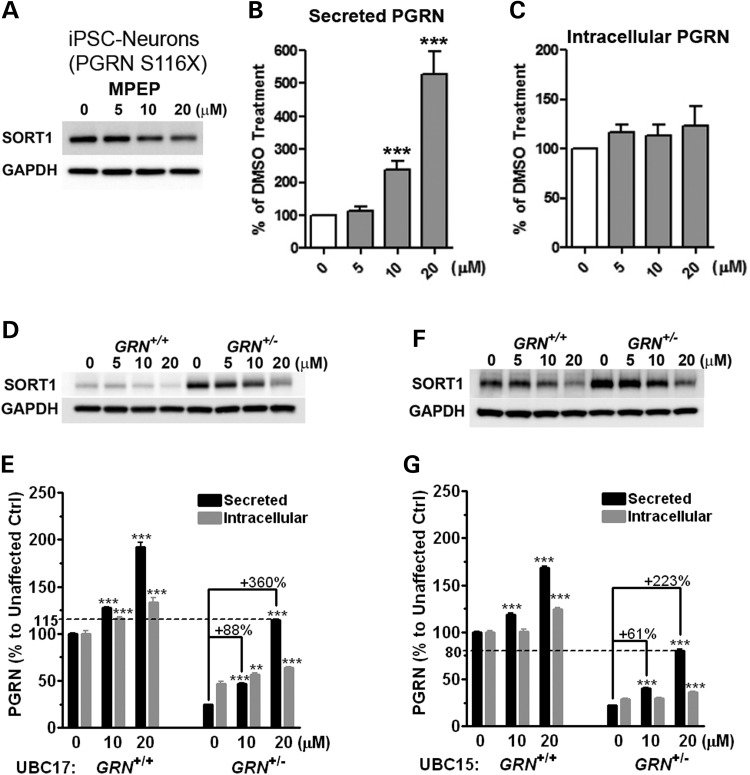
MPEP decreases SORT1 expression and increases extracellular PGRN in cellular models of FTD-*GRN*. (**A**–**C**) MPEP decreased SORT1 levels (A) and increased extracellular PGRN levels (B) but not intracellular PGRN (C) in a recently reported PGRN S116X human neuron model differentiated from FTLD patient-specific iPSCs. (D–G) MPEP reduced intracellular SORT1 levels (D and F) and preferentially increased extracellular PGRN levels (E and G) in lymphoblastoid cell lines (LCLs) from two FTD-*GRN* families, UBC17 (D and E) and UBC15 (F and G). MPEP at 20 μM restored extracellular PGRN to near normal level in mutation carrier (*GRN*^+/−^) compared with non-carrier control (*GRN*^+/+^). **P < 0.01, ***P < 0.001 versus vehicle control, analysis performed by one-way ANOVA followed by Tukey's post-test.

To evaluate whether MPEP counteracts the PGRN haploinsufficiency in the above-mentioned model, the cells were treated with the compound at 5, 10 and 20 μM for 24 h. The result was as follows: in iPSC-neurons carrying a heterozygous PGRN S116X mutation, MPEP treatment dose dependently reduced SORT1 levels (Fig. 2A) and selectively increased exPGRN up to 5.5-fold (Fig. 2B). Furthermore, MPEP treatment had no effect on intracellular PGRN levels (Fig. 2C).

To further evaluate the efficacy of MPEP, we also tested the small molecule on lymphoblastoid cell lines (LCLs) derived from individuals with and without a disease-associated *GRN* mutation. Our investigators initially generated the chosen LCLs, which were first used to validate mutations in *GRN* in FTD patients (3) and were shown to reproduce the PGRN haploinsufficiency phenotype (3,4). Consistent with the results from mammalian cell lines and iPSC-neurons, MPEP treatment decreased SORT1 (Fig. 2D and F) and preferentially increased exPGRN (Fig. 2E and G) in the LCLs from two FTD-*GRN*-affected families: one termed UBC17, which carries a p.C31LfsX34 mutation, and one termed UBC15, which carries a p.R418X mutation (3,4).

In family UBC17 (Fig. 2D and E), 20 μM of MPEP completely restored exPGRN levels in the heterozygous mutation carrier (*GRN*^+/−^-LCL) to a level comparable with the homozygous non-carrier (*GRN*^+/+^-LCL) (Fig. 2E). While MPEP restored exPGRN levels in the UBC15 family to 80% of that of non-carrier *GRN*^+/+^-LCL levels (Fig. 2G), such exPGRN levels represent a 3-fold increase compared with the control. Higher SORT1 levels were also observed in the corresponding UBC17 and UBC15 heterozygous LCL (Fig. 2D and F), which may account for the more severe exPGRN reduction beyond the haploinsufficiency caused by the null mutation (20). At 20 μM, however, MPEP reduced SORT1 levels and increased exPGRN in the *GRN*^+/−^-LCL model to levels comparable with the vehicle-treated *GRN*^+/+^-LCL, indicating a tight correlation between SORT1 reduction and exPGRN induction.

### The PGRN C-terminal motif, PGRN_(589–593)_, is essential for SORT1-mediated endocytosis

**Figure 3.**
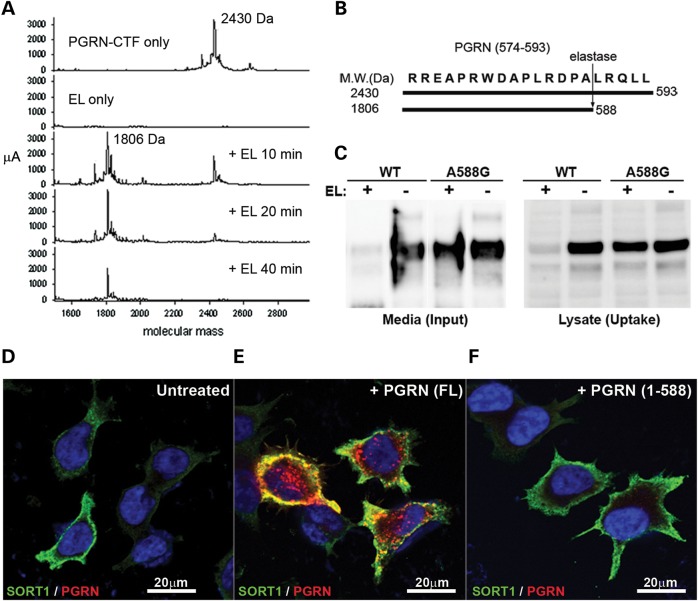
Elastase-mediated removal of C-terminal motif of PGRN blocks PGRN endocytosis by SORT1. (**A**) A synthetic PGRN_574–593_ peptide was enzymatically cleaved by recombinant elastase (EL) in a time-dependent manner as analyzed by MS-MALDI analysis. The PGRN_574–593_ peptide with a molecular weight of 2430 Da was processed into a product with 1806Da indicating the last five residues were removed by EL as shown schematically in (**B**). (**C**) WT or EL-site-mutated A588G-rPGRN protein in cell culture media was set up to react with EL *in vitro*. After reaction, the media was used as input to carry out a cellular endocytosis assay in M17 cells overexpressing SORT1. PGRN endocytosed in cells was detected in cell lysate after treatment (uptake). Unlike WT rPGRN, A588G-rPGRN was more resistant to EL activity as shown by the amount of residual full-length protein in the medium (input), which was endocytosed by cells to a similar extent compared with no EL control. Immunocytofluorescence analysis showed that only full-length rPGRN (FL) (**E**) but not the carboxyl-terminal-truncated PGRN_1-588_ (**F**) was significantly endocytosed by M17^SORT1^ cells. (**D**) The untreated M17^SORT1^ control was included. PGRN and SORT1 were labeled in red and green, respectively.

To identify small molecules that interfere with the SORT1–PGRN interaction via a receptor antagonist approach, first we aimed to locate the specific PGRN region essential for SORT1 binding and endocytosis. Our group and others have shown C-terminal, and not N-terminal, tagging of recombinant human PGRN protein completely blocks intracellular uptake (26) (Supplementary Material, Fig. S2A). In our current study, we identified a crucial neutrophil elastase (NE) cleavage site between residues Ala-588 and Leu-589 of PGRN by *in vitro* digestion of a synthetic PGRN_574–593_ peptide by NE, followed by MALDI-MS analysis (Fig. 3A and B). In addition, we found disruption of the A588 elastase cleavage site, through the introduction of an Ala-to-Gly mutation, protected PGRN^A588G^ from elastase processing, hence preserving the SORT1-interaction motif and PGRN^A588G^ endocytosis (Fig. 3C). To demonstrate that the PGRN residues 588–593 are critical for SORT1-mediated PGRN endocytosis, we produced recombinant truncated PGRN 1–588 (rPGRN_1-588_). The truncated protein failed to be endocytosed in M17 cells overexpressing SORT1 (Fig. 3F), unlike the full-length rPGRN control (Fig. 3E), which presented an endolysosomal localization (Supplementary Material, Fig. S2B), thereby validating the PGRN_589–593_ region as an essential motif for SORT1-mediated PGRN endocytosis.

### Computer-assisted modeling confirms ligands share same SORT1-binding pocket

Identification of small molecules to target protein–protein interaction interfaces is considered extremely challenging owing to the size disparity between small molecules and large contact surfaces on proteins. In addition, protein contact surfaces are usually discontinuous, which further reduces the probability of identifying effective disruptors of protein–protein interactions. To address these challenges in a time-efficient manner, we employed the use of computer-assisted modeling. As our search for a physiological ligand derived from the previously implicated carboxyl-terminus (C-terminus) of PGRN(26) shed new light on the importance of PGRN residues 588–593, we generated models of SORT1 bound to human PGRN_588–593_, neurotensin (NTS), which is a high-affinity SORT1 ligand and mouse Pgrn_584–589_ (-ALRQLL, -ELYENKPRRPYIL and -VPRPLL, respectively).

**Figure 4.**
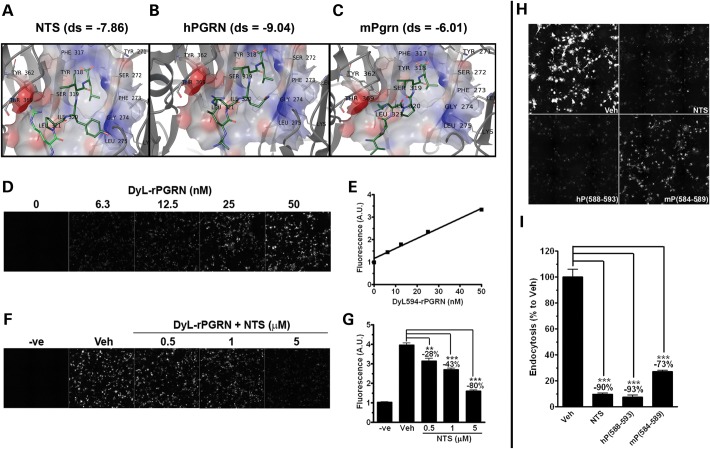
SORT1 ligands competitively inhibit PGRN endocytosis. (**A**–**C**) Structure for NTS (A), native human PGRN_588–593_ peptide (B) and mouse Pgrn_584–589_ peptide (C) docked with SORT1 and the corresponding docking scores are shown. (A) The strongly interacting core of NTS peptide, -PYIL, which is resolved in X-ray structure, is shown in bolder, dark green. SORT1 residues within 4 Å are rendered in gray. Secondary structure for SORT1 is shown as cartoon ribbons. The carboxylate of terminal Leu is visible in the electrostatic surface rendering of the binding pocket. As with NTS, (B) the human PGRN_588–593_ (-ALRQLL) or (C) the mouse Pgrn_584–589_ (-VPRPLL) is shown docked with the SORT1-binding pocket (electrostatic surface rendered) with the residues <4 Å indicated. Carboxylate of Leu is in the identical position as NTS. (**D** and **E**) A quantitative cell-based assay has been established to measure PGRN endocytosis by SORT1. DyLight™ 594-labeled rPGRN was endocytosed dose dependently in COS-1^SORT1^ cells. (D) Images were captured by BD-pathway system. (E) Quantitative cellular endocytosis of DyL-rPGRN was measured by fluorescence signal from treated cells. (**F**) NTS at 0.1, 1 and 5 μM dose dependently inhibited PGRN endocytosis. Untreated COS-1^SORT1^ cells were included as negative control (−ve). (**G**) Quantification of signal from (F). (**H** and **I**) SORT1 ligands at 10 μM, NTS, human PGRN_588–593_ peptide and mouse PGRN_584–589_ peptide competitively inhibited DyL-rPGRN endocytosis as compared with vehicle control, respectively. ***P < 0.001 versus vehicle control, analysis performed either by (G) one-way ANOVA followed by Tukey's post-test or (I) by unpaired student *t*-tests.

We used crystal structure data of SORT1 complexed with NTS(27) to model peptide sequences into the binding cleft of SORT1 using NTS_10-13_ fragment -PYIL as a template, to determine a GRID for docking, and then to optimize the interactions through energy minimization (Fig. 4A–C). The model of the substrate neurotensin (-PYIL fragment) obtains a docked position relative to the crystallographic structure 3F6 K within 0.8 Å RMSD; NTS residues proline and tyrosine fill a largely hydrophobic cleft between the flanking clusters of positive charge, with the NTS Leu side chain embedded in the binding site forming numerous hydrophobic interactions with Phe273, Phe281, Ile294 and Ile320. For each peptide that docked, the carboxylate of the terminal Leu formed strong interactions with SORT1, as shown in Figure 4A–C. The peptides docked with the SORT1-receptor gain stabilization via charge complementarity from electrostatic interactions between the carboxylate anions and Arg292 cations, which is stabilized by Ser283 and Ser319 at carbonyl interactions and bridged by H-bonds. Additional favorable close interactions between the peptides and SORT1 are comprised of hydrophobic and van der Waals interactions and a series of hydrogen bond partners within the binding pocket (Fig. 4A–C). The docking results show NTS with an average docking score of −7.86 kcal/mol (Fig. 4A), human PGRN_588–593_ of −9.041 kcal/mol (Fig. 4B) and mouse Pgrn_584–589_ of −6.85 kcal/mol (Fig. 4C). As such, the human PGRN_588–593_ has the highest binding affinity followed by NTS and then the mouse Pgrn_584–589_. Higher-affinity binding of human PGRN_588–593_ peptide to SORT1 compared with that of NTS/SORT1 is derived from strong interaction pairs between the terminal few residues and key side chains and backbone interactions from SORT1. Additionally, the mouse mPgrn_584–589_/SORT1 binding suffers a loss in interaction from a distinct repositioning of mPgrn_584–586_ residues, -VPR, without significant SORT1 contacts. The details of the docking models and decomposition of individual interactions between SORT1 and NTS, human PGRN_588–593_ and mouse Pgrn_584–589_ are described in the Supplementary Material.

### SORT1 ligands competitively inhibit full-length PGRN endocytosis

Given our computer-assisted modeling confirmed and provided new information of how NTS (28,29) and PGRN_588–593_ share a similar SORT1-binding site (19), we sought to determine whether the use of these ligands as antagonists could be an effective strategy to inhibit PGRN endocytosis in cell culture. Using DyLight™-594 fluorescence-labeled rPGRN (DyL-rPGRN) and SORT1-expressing COS-1 (COS-1^SORT1^) cells, we established a cell-based assay to quantitatively analyze PGRN endocytosis by measuring DyLight™-594 emission from endocytosed DyL-rPGRN upon treatment. Addition of titrated amounts of DyL-rPGRN (0 to 50 nM) to COS-1^SORT1^ cells produced a linear, non-saturated endocytosis response (Fig. 4D and E). As expected, co-treatment of DyL-rPGRN with NTS (i.e. 0.5, 1 and 5 μM) dose dependently inhibited PGRN endocytosis (Fig. 4F and G). At the same concentrations, NTS, as well as human PGRN_588–593_ and mouse PGRN_584–589_, respectively, inhibited full-length PGRN endocytosis by 90, 93 and 73% (Fig. 4H and I). The fact that the mouse ortholog peptide of PGRN_588–593_ also significantly inhibited full-length PGRN endocytosis by human SORT1 in COS-1^SORT1^ cells, albeit to a lesser extent, suggests that the PGRN-CT motif is evolutionarily conserved. In a protein co-immunoprecipitation assay, we further validated that human PGRN_588–593_ peptide, NTS and mouse Pgrn_584–589_ peptide can each inhibit the physical interaction between full-length PGRN and SORT1 (Supplementary Material, Fig. S3). These results support the notion that the development of SORT1 antagonists, such as stabilized forms of chemically modified human PGRN_588–593_ peptide or NTS mimetics, may serve as potential exPGRN enhancers through inhibition of extracellular clearance.

### Use of small-molecule and antibody binders of the PGRN_588–593_ motif inhibit SORT1-mediated PGRN endocytosis

**Figure 5.**
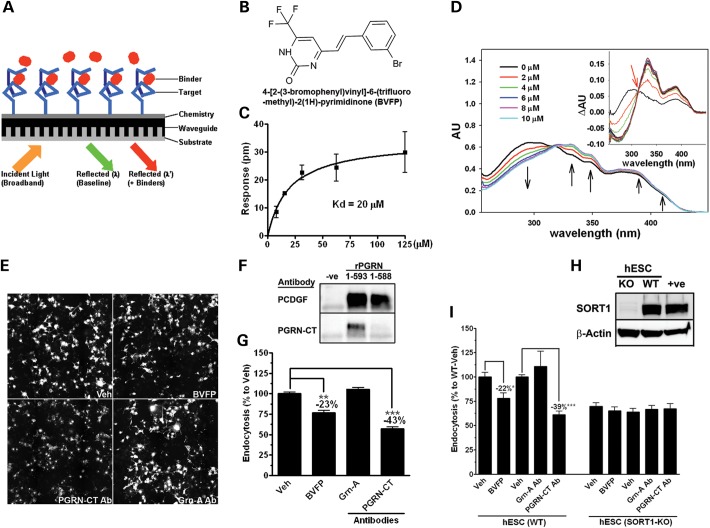
Small-molecule and antibody binders of the PGRN_588–593_ motif inhibit SORT1-mediated PGRN endocytosis. (**A**) Schematic diagram illustrating the detection mechanism of Epic^®^ biochemical binding assay. When screening for compound binders of PGRN_588–593_ peptide (target), the specific binding events are detected by changes in reflected resonant wavelength before (λ) and after (λ′) addition of a potential compound binder. (**B**) Structure and chemical name of BVFP, an identified compound that binds the PGRN_(588–593)_ peptide. (**C**) Saturated binding curve of BVFP to PGRN_(588–593)_ peptide (*n* = 4). (**D**) Wavelength red-shifting of UV-absorption spectra of BVFP (20 μM) upon titration with the PGRN_(588–593)_ peptide. The interaction between BVFP and the peptide is represented by distinct changes in the absorption intensities (black arrows). The presence of an isosbestic point at 313 nm (inset red arrow) also confirmed the interactions. (**E**) The PGRN_(588–593)_ binders, BVFP (5 μM) and PGRN-CT antibody (80 nM) inhibited rPGRN endocytosis as tested by the quantitative cell-based assay in COS-1^SORT1^ cells. Both binders were pre-incubated with rPGRN_1-593_ for an hour and then added to the cells for an hour to allow endocytosis. A GRN-A specific antibody (80 nM) was used as a negative control. (**F**) Vehicle control (−ve), full-length protein (rPGRN_1-593_) or truncated protein (rPGRN_1-588_) was analyzed by western blot using PCDGF or PGRN-CT antibody for detection. The PGRN-CT antibody was confirmed to be PGRN_(588–593)_ dependent. (**G**) Fluorescence signal quantification of (E). (**H**) Western blot analysis confirmed the absence of SORT1 protein in the SORT1^KO^-hESCs. (**I**) BVFP and the PGRN-CT antibody inhibited PGRN endocytosis in WT-hESCs but not in SORT1^KO^-hESCs indicating that the effect was SORT1 dependent. *P < 0.05, **P < 0.01, ***P < 0.001 versus vehicle control, analysis performed by unpaired student *t*-tests.

Given that restoring exPGRN levels using SORT1 antagonists can potentially trigger off-target effects related to NTS (29), lipoprotein lipase (30) and LDL-receptor-associated protein (31) function and cause undesirable clinical side-effects, modulating the PGRN–SORT1 interaction via PGRN-specific binders may be a promising approach. To identify small molecules that can selectively bind to the PGRN C-terminal motif to inhibit PGRN–SORT1 interactions, we screened 4800 compounds from a commercial library against a custom-synthesized PGRN_588–593_ peptide using a 384-well format, biochemical binding assay that utilizes resonant waveguide grating biosensor detection (Fig. 5A) (Supplementary Material, Method). We identified a small molecule, 4-[2-(3-bromophenyl)vinyl]-6-(trifluoromethyl)-2(1H)-pyrimidinone termed BVFP (Fig. 5B), that binds to the PGRN_588–593_ peptide at a low micromolar concentration (*K*_d_ = 20 μM, Fig. 5C). Upon mixing and titrating BVFP with the PGRN_588–593_ peptide, we detected a shift in the ultraviolet(UV)-absorption spectra of BVFP and distinct changes of absorption intensities, which in addition to the presence of an isosbestic point at 313 nm, validated the BVFP and PGRN_588–593_ peptide interaction (Fig. 5D).

To determine whether BVFP, when bound to PGRN, disrupts full-length PGRN and SORT1 binding, we devised a SORT1-dependent rPGRN precipitation assay utilizing the Meso-Scale Discovery (MSD) system (Supplementary Material, Fig. S4A and B). Immobilized SORT1 generated a specific binding signal from the amino-terminal-tagged PGRN (N-rP) but not from the C-terminal-tagged PGRN, validating once again the PGRN–SORT1 binding is C-terminal dependent. A dose-dependent inhibition of rPGRN binding to immobilized SORT1 was observed in the presence of 20 and 200 μM of BVFP, respectively, proving the small molecule's capacity to disrupt PGRN–SORT1 binding (Supplementary Material, Fig. S4C). To include a higher-affinity PGRN_588–593_ motif binder as a positive control, an antibody targeting the motif, PGRN-CT antibody, was made for validating the approach (Fig. 5F). Using our quantitative endocytosis assay (Fig. 5E and G), we found that BVFP inhibited DyL-rPGRN endocytosis by 23% when treated at 5 μM, compared with 43% inhibition by PGRN-CT antibody when treated at 80 nM (Fig. 5E and G). Note that a negative control antibody targeting GRN-A had no effect on DyL-rPGRN endocytosis. To show that the effects of BVFP and the PGRN-CT antibody are SORT1 dependent, the PGRN endocytosis experiment was repeated in human embryonic stem cell lines (hESCs) with and without SORT1 expression established by using the transcription activator-like nucleases genome-editing system (TALENs) (32). First, we found SORT1 was abundantly expressed in the wild-type hESCs (Fig. 5H). Similar to the results from COS-1^SORT1^ cells, BVFP and PGRN-CT antibody inhibited endocytosis of DyL-rPGRN by 22 and 39%, respectively, under the same experimental conditions in wild-type hESCs (Fig. 5I left). Treatment of DyL-rPGRN to the isogenic hESCs-SORT1^KO^ line, which has an identical genetic background and epigenetic state compared with the parent hESC-WT line, generated a significantly lower signal compared with the WT-hESCs, indicating impaired PGRN endocytosis in the absence of SORT1 (Fig. 5H). Both BVFP and PGRN-CT antibody had no effect on DyL-rPGRN endocytosis in the hESCs-SORT1^KO^ line (Fig. 5I right), confirming that their effect on PGRN endocytosis is SORT1 dependent.

## DISCUSSION

Given that partial loss of PGRN, owing to mutations in the PGRN gene (*GRN*), is causative of FTLD with TDP-43 pathology (i.e. FTLD-TDP), therapeutically restoring PGRN levels may be a promising therapeutic strategy. In fact, evidence from numerous studies suggests that PGRN acts as a protective neurotrophic factor by regulating neuronal survival and neurite growth in cortical/motor neurons, immortalized cell lines and zebra fish (33–35) and that PGRN is protective against insults induced by TDP-43 (36). Given that there is currently no feasible way to pharmacologically manipulate PGRN levels in the brain and that recombinant PGRN is too large to cross the blood–brain barrier (BBB) for protein replacement, the development of bio-available, BBB permeable PGRN enhancers will be a valuable tool to determine whether therapeutically modulating or increasing PGRN levels can alleviate the pathogenesis associated with FTD-*GRN*.

While new strategies designed to upregulate PGRN levels have emerged, challenges remain in terms of limiting off-target effects. For example, Cenik *et al*. recently identified the histone deacetylase (HDAC) inhibitor suberoylanilide hydroxamic acid as an enhancer of *GRN* expression (18). Christian Haass' group also has recently demonstrated four independent and highly selective inhibitors of vacuolar ATPase (V-ATPase) significantly elevate intracellular and secreted PGRN (17). However, because HDAC and V-ATPase inhibitors can potentially affect a wide-range of genes at transcriptional and post-translational levels, respectively, thereby increasing the likelihood that the inhibitors will also trigger undesirable, clinical side-effects and liabilities, the development of a strategy to improve target specificity may hold more promise.

**Figure 6.**
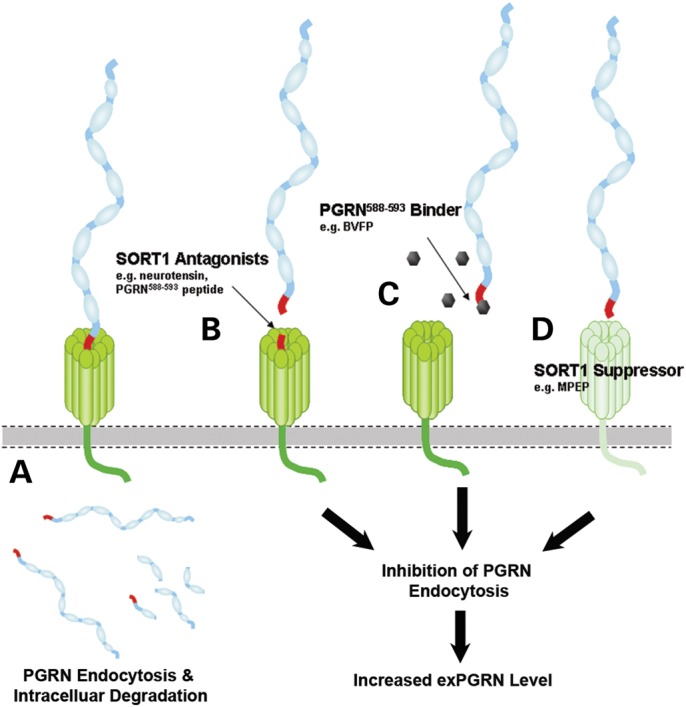
Schematic diagram summarizing the strategies applied to inhibit SORT1-mediated endocytosis in the current study. (**A**) Under normal conditions, extracellular PGRN interacts with the β-propeller tunnel structure of SORT1 using its C-terminal end binding motif as shown in red color. SORT1 facilitates endocytosis of exPGRN and directs it to the endolysosomal pathway for degradation. (**B**) High-affinity SORT1 ligands such as NTS or the PGRN_(588–593)_ peptide competitively limits the access of exPGRN to SORT1-binding sites, thereby inhibiting PGRN endocytosis. (**C**) To improve target specificity, we have also identified a small-molecule binder, BVFP, targeting the PGRN_(588–593)_ motif that is essential for PGRN–SORT1 interaction. We demonstrated that pretreatment of BVFP to rPGRN significantly reduced the amount of rPGRN captured by SORT1 *in vitro* and inhibited SORT1-mediated rPGRN endocytosis. (**D**) Suppressors of SORT1 expression, such as MPEP, reduce SORT1-mediated endocytosis, thereby increasing extracellular PGRN levels. The above-mentioned strategies, used alone or in combination with others, are potential avenues for discovery of SORT1-dependent PGRN enhancers for the treatment of FTD-*GRN*.

Here, we demonstrate the preclinical efficacy of several approaches through which impairing PGRN's interaction with SORT1, a neuronal receptor that mediates PGRN endocytosis and degradation, restores extracellular PGRN levels in FTD patient-derived iPSC-neurons and lymphocytes (Fig. 6). To the best of our knowledge, our report is the first to demonstrate the efficacy of enhancing PGRN levels in iPSC-neurons derived from FTD patients with PGRN deficiency. We also validate that the bioactive compound MPEP preferentially increases exPGRN levels by suppressing or reducing intracellular SORT1 levels without affecting sortilin-related family members SORLA and SORCS1. To understand whether MPEP might increase exPGRN through protease(s) inhibition, we performed a database search using the similarity ensemble approach tool (37). No protease inhibitors in the database were found to have significant structural similarity to MPEP, suggesting that mechanisms other than protease inhibition account for the SORT1-targeted exPGRN upregulation by MPEP.

Given SORT1′s role in regulating exPGRN levels, we further demonstrate that SORT1 antagonists and PGRN_588–593_ binders inhibit SORT1-mediated PGRN endocytosis. Our novel cell culture data reveal the PGRN_588–593_ peptide is the critical region for SORT1–PGRN binding; the ‘hot spot’ is confirmed by our computational model of the PGRN–SORT1 interaction. As mentioned, the feasibility of finding a small molecule that specifically targets protein–protein interactions is very much case dependent(38), but identifying the crucial site improved our ability to hone in on high-affinity, small-molecule binders targeting either binding partner. To reduce undesirable effects on other SORT1 ligands, we concentrated our efforts on a compound library screen and biochemical binding assay that allowed us to identify a small molecule, BVFP, that targets the C-terminal PGRN_588–593_ motif. As the motif is located on a linker region and not on the functional granulin regions, binding of BVFP to the extreme C-terminal motif is unlikely to significantly impair PGRN function (1). Moreover, the discovery of a potential NE cleavage site at the C-terminal Ala-588 residue of PGRN suggests that a surge in extracellular NE levels would increase C-terminal truncation at Ala-588 leading to accumulation of the PGRN_1-588_ protein and granulins generated by increasing NE proteolytic activity. As NE is an innate immune response mediator that triggers downstream molecular events required for the inflammation process (39), the Ala-588 site is likely serving as a regulatory mechanism to boost extracellular PGRN and granulins levels that are involved in the inflammation signaling cascade.

While we demonstrate BVFP prevents SORT1-medited endocytosis and increases exPGRN levels, an alternate supplementary approach would be to identify small molecules targeting SORT1, specifically at the Arg-292-, Ser-283- and Ser-319-binding pockets. A structure-based virtual high-throughput screening approach (vHTS) using the available NTS-complexed SORT1 crystallographic data could be employed to accelerate drug discovery (27). The vHTS hits can be validated by a functional assay such as the quantitative endocytosis assay described in the current study.

We also evaluated the use of high-affinity SORT1 ligands as competitive inhibitors to effectively block exPGRN's access to SORT1′s binding site. Our computer-assisted modeling sheds new light on why we observed a dramatic reduction in PGRN endocytosis when ligands, such as NTS and human PGRN_588–593_, were present *in vitro*: the binding pocket and protein–protein interactions of NTS and PGRN's C-terminal binding motif significantly overlap, hence explaining the competitive inhibition effect. Although both NTS and full-length PGRN induce endocytosis upon binding to SORT1, the two ligands have been reported to traffic to different subcellular localizations, namely endolysosomal compartment (19) and the trans-Golgi network (40). In both cases, SORT1 is dissociated from the bound ligands in early endosomes and then recycled back to the cell surface to drive the endocytosis process. While the use of a SORT1 antagonist approach is enticing owing to affinity for and efficacy in blocking PGRN uptake, this approach is also more likely to trigger off-target effects by disrupting binding between SORT1 and other ligands that occupy a similar binding pocket.

As with many other ligands such as prosaposin (41), acid sphingomyelinase (42) and cathepsins D and H (43), evidence suggests SORT1 is a sorting receptor of PGRN for lysosome delivery and degradation (19). Our group (5) and another (21) also showed that SORT1 is not essential for stimulating neurite outgrowth in cultured neurons, suggesting that SORT1 might not be involved in the PGRN-mediated signaling for neurotrophic function. Nevertheless, inhibition of PGRN endocytosis might influence PGRN's intracellular functions or unidentified intracellular signaling pathways, especially in cells that rely on its uptake from the extracellular space or neighbor cells. It is known that complete deficiency in PGRN is a cause of clinical neuronal ceroid lipofuscinosis (NCL) (44), a rare lysosomal storage disease characterized by intraneuronal accumulation of autofluorescence lipofuscins. Based on this finding, it is reasonable to speculate that PGRN has a lysosomal function related to turnover of lipofuscin-related substrates. While blocking PGRN endocytosis will reduce level of PGRN in lysosomes, intracellular PGRN provided from the biosynthetic pathway to lysosomes is expected to be enough to maintain normal lysosomal function. At least for most lysosomal storage disorders, clinical onset is often associated with >90% loss of activity of the corresponding enzyme (45). However, further studies will be required to understand whether abrogation of PGRN endocytosis can cause NCL or other deleterious biological consequences.

Given the challenges associated with the development of therapies and clinical trials for patients suffering from neurodegenerative diseases, the need is urgent and the time is now for the medical community to rally behind a promising target. The increased excitement surrounding PGRN-based therapies is warranted: the specific patient population that could benefit is easily identifiable thanks to genetic testing. Moreover, the feasibility of restoring PGRN to normal levels has been shown by our group and others. The fact that genetic ablation of *SORT1* restores and normalizes PGRN levels in brain uniquely positions the SORT1–PGRN axis as an ideal target for PGRN-based therapy in FTD-*GRN* (19). Herein, we validated multiple strategies to target the SORT1–PGRN axis with the aim of enhancing PGRN levels. We show that SORT1 antagonists and PGRN_588–593_ binders inhibit SORT1-mediated PGRN endocytosis and a SORT1 suppressor restores exPGRN in patient-derived cell models. In addition, both small molecules identified in this study, namely MPEP and BVFP, are in compliance with the Lipinski's Rule of five to predict drug-likeness properties, which should facilitate *in vivo* studies in the near future. Further studies to understand the dosage effect of PGRN-enhancing reagents to treat FTD-*GRN* will be essential, given neutralizing PGRN to counteract its possible tumorigenic effect (46) is a proposed cancer treatment (47,48). In addressing such concerns, and further exploring the relationship between TDP-43, SORT1 and PGRN (49), the clinical development of PGRN-based therapy for FTD-*GRN* patients will and should continue.

## MATERIALS AND METHODS

### Recombinant PGRN protein, plasmids and antibodies

Wild-type or mutant human recombinant PGRN protein (rPGRN) with a 6-histidine tag on the amino-terminus (N-rP) or the carboxyl-terminus (C-rP) was purified from culture media of stable HEK293 cells secreting the corresponding protein. Purification procedures were the same as previously published (5). All the plasmids used in this study are listed as follows: pCDNA4-PGRN(1–593) (N-terminal 6His tag), pCDNA4-PGRN(1–588) (N-terminal 6His tag), pCMV-SORT1 (OriGene), pCDNA6-SORT1 (C-terminal Flag tag) and pEGFP-N1 (Clontech). All the antibodies used in this study are listed as follows: PCDGF (or Grn-E specific) antibody (1:1000 for western blot) (Invitrogen), human PGRN monoclonal antibody (1:200 for immunofluorescence) (R&D Systems), Grn-A antibody (80 nM for blocking PGRN endocytosis) (Novus; 26320002), PGRN-CT antibody (1:1000 for western blot; 80 nM for blocking PGRN endocytosis) (21st Century; affinity purified rabbit polyclonal immunized with PGRN_574–593_ peptide), sortilin antibody (1:3000 for western blot; 1:1000 for immunofluorescence) (Abcam; ab16640), SORLA antibody (1:200) (R&D Systems; AF5699), SORCS1 antibody (1:1000) (R&D Systems; AF3457), ubiquitin antibody (1:1000) (DAKO; Z0458), EEA1 antibody (1:100 for immunofluorescence) (BD Biosciences; 610457), giantin antibody (1:1000 for immunofluorescence) (Abcam; ab24586), Flag M2 monoclonal antibody (1:500 for immunoprecipitation) (Sigma), GAPDH antibody (1:10 000 for western blot), and β-actin antibody (1:10 000 for western blot).

### Western blot analyses

PGRN, SORT1, GFP, GAPDH and β-actin proteins were analyzed using 10% Tris–glycine polyacrylamide gel by SDS–PAGE. After electrophoresis, proteins on gel were transblotted to PVDF membranes followed by standard immunoblotting protocols.

### Analysis of PGRN endocytosis in M17^SORT1^ cells

M17 cells seeded in 12-well culture plates (western blot) or on glass coverslips in 24-well culture plate (immunofluorescence) were transfected with pEGFP-N1 (Clontech) or pCMV-SORT1 (OriGene) vectors. Next day, the cells were changed into serum-free medium 1 h before addition of rPGRN (500 ng/ml) and treated for 60 min. The cells were then put on ice and washed with cold PBS. For western blot, cells were lysed in cold M-Per protein extraction buffer followed by standard procedures as mentioned. For immunofluorescence analyses, cells were fixed in 4% paraformaldehyde solution followed by PBS washes. The cells were then incubated in blocking buffer (PBS, 5% goat serum, 0.1% saponin) for 1 h at room temperature followed by incubation in primary antibody overnight at 4°C. The cells were incubated with secondary antibodies (1:1000) for 1 h at room temperature. The coverslips were then mounted onto slides and waited overnight before analysis. Image analysis was performed by Zeiss LSM 510 META confocal microscope using 60× magnification setting.

### MALDI-MS analysis of NE-processed PGRN_(584–593)_ peptide

An enzymatic reaction was set up with 7 μg of custom-synthesized PGRN_(584–593)_ peptide (Mayo peptide synthesis core) and 20 μg of NE in a 25 μl reaction at 37°C. Every 10 min, 4 μl of the mixture was collected and diluted 10 times in a solvent mixture (50% acetonitrile:50% water:0.1% acetic acid) until 1 h had passed. For MALDI-MS analysis, 1 μl of the diluted reaction mixture was dried on a MS gold chip and then layered with a concentrated sinipinic acid matrix on top for crystallization. Afterwards, the samples were analyzed by the Bio-Rad MALDI-MS system.

### Quantitative endocytosis cell-based assay

COS-1 cells seeded on a 96-well black plate 1 day before were transfected with pCMV-SORT1 vector. After 24 h, cells were treated with fluorescence-tagged rPGRN (DyL-rPGRN) pre-labeled by DyLight™ 594 antibody labeling kit (Thermo Scientific). The DyL-rPGRN was diluted in OptiMEM to tested concentrations and incubated with the cells for 1 h for endocytosis. The cells were then washed with cold PBS and then fixed by 4% paraformaldehyde. After washing twice, each well was filled with PBS prior scanning. The total PGRN fluorescent signal from the cells was obtained by a plate reader with 593/618 nm (Ex/Em) settings. The endocytosis signal was normalized by the total nuclei signal obtained by staining with a Hoechst 33342 dye. Baseline endocytosis signal was defined as a signal from medium-only-treated cells, whereas the 100% endocytosis level was set as the signal obtained from cells treated with 50 nM DyL-rPGRN. For testing SORT1 ligands, DyL-rPGRN and the peptide were added simultaneously to the cells. For testing of PGRN binders, the binder was pre-incubated with DyL-rPGRN for 1 h before adding to the cells. For qualitative analysis, images from each well were captured by BD Pathway 855 system using a 20× magnification setting.

### PGRN co-immunoprecipitation

HEK293T cells were transfected empty vector or pCMV-SORT1-Flag vector for 48 h. Then, cells were lysed by using Co-IP buffer. Pre-incubation was performed with 300 μg of lysate protein mixed with 20 μM NTS, human PGRN_(588–593)_ peptide or mouse PGRN_(584–589)_ peptide for 1 h. Then, rPGRN (100 nM) was added into the protein G beads pre-cleared supernatant and mixed for 30 min. Next, anti-Flag M2 agarose (Sigma) was added and mixed for another 4 h. The agarose was collected by centrifugation at 1000 g for 3 min and washed with Co-IP buffer six times. Captured protein was eluted from the beads using loading buffer and analyzed by western blot.

### Determination of UV-absorption spectrum of BVFP

Stock solutions of compound BVFP (20 mm) and PGRN_(588–593)_ peptide (12.96 mm) were prepared in DMSO and water, respectively. For the UV absorption experiment, both components were diluted to give final concentrations of 20 μM BVFP and 324 μM peptide. The interaction between BVFP and the PGRN_(588–593)_ peptide was monitored by scanning over the UV absorbance range of 200–800 nm on a Cary 3 Bio UV–visible spectrophotometer (Varian), as a small amount of KKGRN peptide (0.1 equivalence/1 μl per addition) was titrated into the 20 μM BVFP sample. In addition to baseline correction with DMSO, a control titration experiment using water in place of the PGRN_(588–593)_ peptide was performed under identical conditions in order to correct for potential spectral changes owing to sample dilution.

### iPSC neuronal culture

An induced pluripotent stem cell (iPSC) line from an FTD patient carrying a *GRN* nonsense mutation (p.S116X) was differentiated into neurons as described previously (22). Two-week-old neurons were incubated with 5–20 µM MPEP or DMSO (in fresh culture medium) for 24 h. The medium was then collected, and cells were lysed with 1% NP-40 lysis buffer. PGRN levels in total cell lysates and culture medium were determined with an ELISA kit (Alexis Biochemicals, San Diego, CA, USA) according to the manufacturer's instructions.

### MSD system-based immunoassay to analyze human PGRN

The assay was performed according to manufacturer's instructions. A goat anti-PGRN antibody (R&D Systems) was used as capture antibody. PGRN standards (from 0.5 to 50 ng/ml) were from purified N-6His-rPGRN protein. For detection, a 1:1 ratio of an anti-PCDGF antibody (Invitrogen) and a SULFO-TAG-labeled anti-rabbit IgG antibody were applied. The MSD Sector Imager 2400 was used to read assay signal.

### A MSD-based SORT1–PGRN interaction assay

M17 cells were transfected with pCMV-SORT1-Flag for 48 h. Then, the cell lysate was harvested by the same method used in the co-immunoprecipitation assay. Expression of SORT1-Flag protein was confirmed by western blot. MSD plate was first coated with the Flag M2 antibody (Sigma). Following an overnight incubation at 4°C, the plate was blocked for 2 h and then washed prior to addition of 5 µg/well of lysate protein containing SORT1-Flag protein. After overnight incubation at 4°C, it was then washed and added with 200 nM of either N-6His- or C-6His-tagged rPGRN pre-incubated with either vehicle, 20 µM or 200 µM of BVFP, for 30 min at room temperature. Then plate was then incubated for 2 h, washed and incubated with a mixture of PCDGF antibody and a SULFO-TAG-labeled anti-rabbit IgG antibody for 2 h. After final wash, the plate was read by the MSD Sector Imager 2400.

### Quantitative reverse transcription–polymerase chain reaction

Quantitative PCR (qPCR) was performed using an HT7900 analyzer (Applied Biosystems, Carlsbad, CA, USA). Each 5 μl reaction contained: 2 μl of pre-diluted (1:50) cDNA, 0.5 μl primer mix (1 μM for each primer) and 2.5 μl SYBR green (Invitrogen). The thermal cycling conditions were as follows: 50°C for 2 min, 95°C for 2 min, 40 cycles of 95°C for 15 s and 60°C for 1 min. The mRNA levels of SORT1 and PGRN were normalized to GAPDH, an endogenous transcript control. The data were analyzed by using Software RQ Manager 1.2 (Applied Biosystems). Primer sequences for amplification of PGRN were 5′ CCCTTCTGGACAAATGGC 3′ and 5′ GGAAGTCCCTGAGACGGTAA 3′. Primer sequences for GAPDH were 5′ CTTTGGTATCGTGGAAGGACTCATG 3′ and 5′ CCAGTAGAGGCAGGGATGATGTTC 3′. Primer sequences for SORT1 were 5′ GGCCTGTGGGTGTCCAAGAA 3′ and 5′ GCAGGAGCCATTTGCATAGGTT 3′.

### Statistical analysis

Representative data of at least three independent experiments with reproducible results were shown. All statistical analysis was performed by unpaired student *t*-test. One-way analysis of variance (ANOVA) followed by Tukey's post-test was applied in multi-doses experiments.

## SUPPLEMENTARY MATERIAL

Supplementary Material is available at *HMG* online.

## FUNDING

This work was supported by Mayo Clinic Foundation (L.P.), NIHR01 AG026251, R01 NS 063964-01, R01 NS077402, R01 ES20395-01, R21 NS84528-01J (L.P.), NS065782 (R.R.), NIH R01 AG026251, R01 NS057553 (F.B.G.) and the Consortium for Frontotemporal dementia Research (R.R.) (F.B.G.). Funding to pay the Open Access publication charges for this article was provided by the Mayo Clinic.

## Supplementary Material

Supplementary Data
