# Supplementary material for: Targeted manipulation of the sortilin–progranulin axis rescues progranulin haploinsufficiency
Source: Hum Mol Genet. 2013 Oct 26;23(6):1467–78. doi: 10.1093/hmg/ddt534 (PMC3929086; doi:10.1093/hmg/ddt534)
Supplement: Supplementary Data [file supp_23_6_1467__index.html]

Targeted manipulation of the sortilin-progranulin axis rescues progranulin haploinsufficiency — Targeted manipulation of the sortilin–progranulin axis rescues progranulin haploinsufficiency — Targeted manipulation of the sortilin–progranulin axis rescues progranulin haploinsufficiency — Supplementary Data 

# Targeted manipulation of the sortilin–progranulin axis rescues progranulin haploinsufficiency

## Supplementary Data

Supplementary Data

**Files in this Data Supplement:**

- Supplementary Data - Pdf file
