# Supplementary material for: Targeted manipulation of the sortilin–progranulin axis rescues progranulin haploinsufficiency
Source: Hum Mol Genet. 2013 Oct 26;23(6):1467–78. doi: 10.1093/hmg/ddt534 (PMC3929086; doi:10.1093/hmg/ddt534)
Supplement: Supplementary Data [file supp_ddt534_ddt534supp.pdf]

SUPPLEMENTARY FIGURE 1

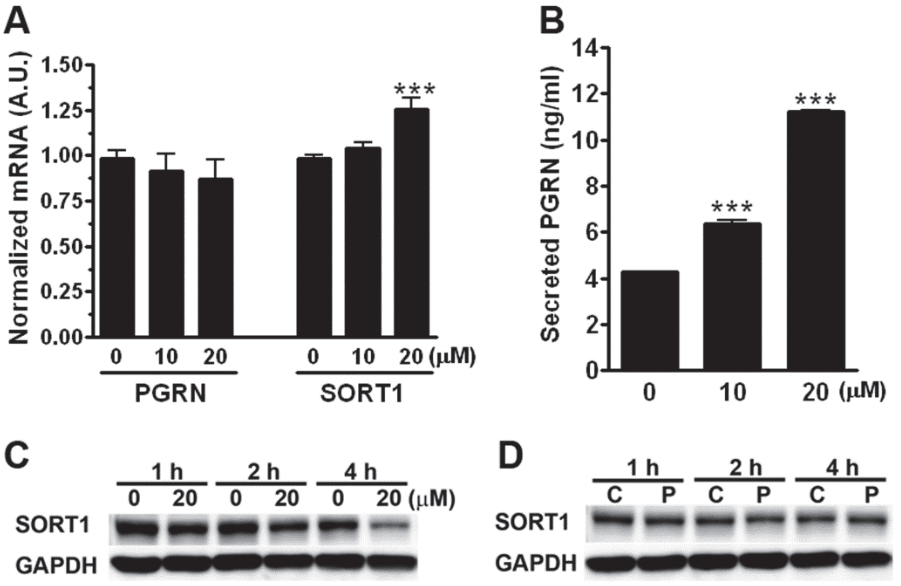

MPEP reduces SORT1 protein levels by a transcription-independent mechanism. **(A)** *PGRN* and *SORT1* mRNA levels in MPEP-treated M17 cells were analyzed by real-time quantitative PCR. No significant changes in *PGRN* or *SORT1* mRNA were detected, except for a mild increase in *SORT1* mRNA at 20 μM of MPEP treatment. In each case, the levels of mRNA were normalized to *GAPDH* mRNA. **(B)** In the same experiments, culture media were analyzed for secreted PGRN and confirmed the induction of PGRN secretion by MPEP. **(C)** M17 cells were treated with MPEP at 20 μM for 1, 2 and 4 hours. Significant reduction of SORT1 induced by MPEP treatment was detected beginning at 2 hours post-treatment. **(D)** M17 cells were treated with control (C) or rPGRN (P) at 50 ng/ml for 1, 2 and 4 hours. No significant changes in SORT1 level were detected under these conditions suggesting that MPEP-induced SORT1 down-regulation is independent of autocrine regulation exerted by increased extracellular PGRN level. *** $P < 0.001$ vs. vehicle control, analysis performed by One-way ANOVA followed by Tukey post-test.

SUPPLEMENTARY FIGURE 2

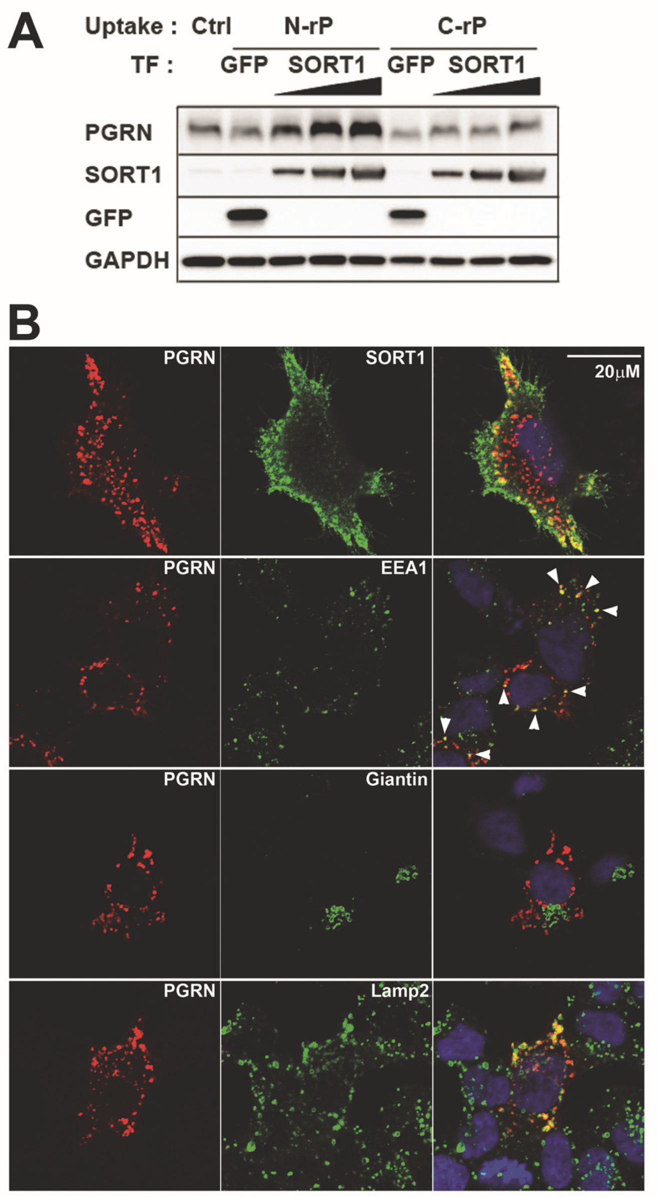

SORT1-dependent PGRN endocytosis in M17 neuroblastoma cells. **(A)** SORT1 dose-dependently endocytosed amino-terminal 6-His tagged rPGRN (N-rP), but not C-terminal 6-His tagged rPGRN (C-rP) to the intracellular fraction in M17 cells expressing titrated amounts of SORT1. M17 cells expressing GFP were used as negative control. The amount of PGRN, SORT1, GFP and GAPDH in cell lysates were analyzed by Western blot. **(B)**

Immunocytofluoescence analysis of SORT1-mediated PGRN endocytosis showed that N-rP was paritally colocalized with exogenous SORT1 near the plasma membrane, endogenous EEA1 (early endosome marker) and endogenous LAMP2 (lysosome marker) but not with endogenous giantin (golgi-apparatus marker).

SUPPLEMENTARY FIGURE 3

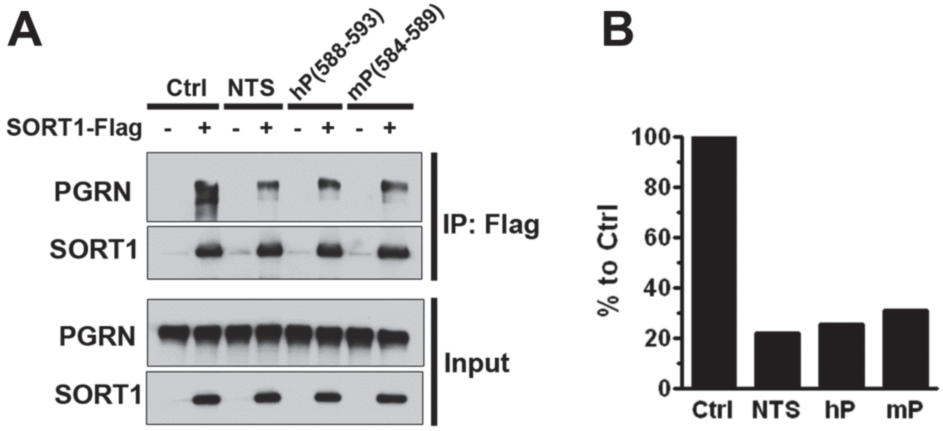

SORT1 antagonists inhibit PGRN-SORT1 interactions in a co-immunoprecipitation assay. **(A-B)** HEK293 cell lysate expressing Flag-tagged SORT1 was incubated with 100 nM of rPGRN in the presence of vehicle (Ctrl), neurotensin (NTS), PGRN(588-593) peptide or mPgrn(584-589) peptide, all at 10 μM. Using anti-Flag M2 agarose, SORT1 proteins and the bound rPGRN were immunoprecipitated (IP, top panel) and subsequently analyzed by Western blot **(A)** and quantified by densitometry **(B)**. All three peptides significantly reduced the amount of rPGRN that immunoprecipitated with SORT1, indicating the inhibition of rPGRN/SORT1 protein-protein interactions.

SUPPLEMENTARY FIGURE 4

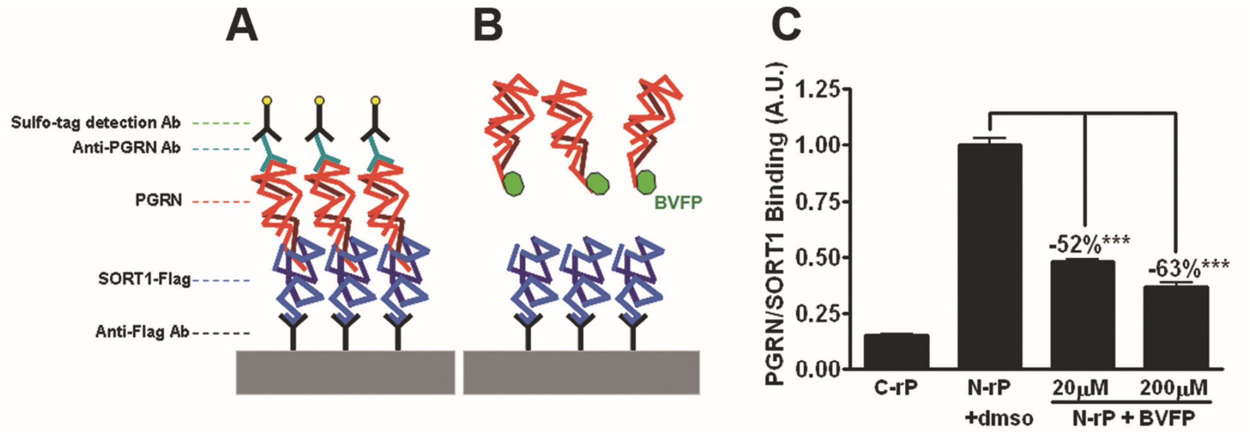

The $PGRN_{588\text{-}593}$ binder BVFP inhibits PGRN-SORT1 interactions. **(A)** Schematic diagram illustrating the components of the PGRN-SORT1 binding assay. M17 cell lysates overexpressing Flag-tagged human SORT1 protein is added to MSD-assay plated coated with anti-Flag antibody. Immunocaptured SORT1-Flag protein on a MSD-assay plate is used as bait to precipitate PGRN. Detection and quantification of precipitated PGRN is then measured by using an anti-PGRN antibody and a sulfo-tag detection antibody. **(B)** BVFP binding to the $PGRN_{588\text{-}593}$ motif may prevent PGRN from interacting with SORT1 therefore reducing the amount of PGRN captured. **(C)** Pre-incubation of BVFP with the N-terminal 6His-tag rPGRN (N-rP) significantly reduced the amount of PGRN that immunoprecipitated with SORT1. Note that a C-terminal 6His-tag rPGRN (C-rP) group was included as a negative control. *** $P < 0.001$ vs. vehicle control, analysis performed by One-way ANOVA followed by Tukey post-test.

SUPPLEMENTARY METHODS AND RESULTS

Epic(R) biochemical assay and compound library screen

Essentially, the Epic® is a spectrophotometric reader that uses a resonant waveguide grating detection mechanism to detect biochemical interactions (Fig.5A). In brief, the waveguide grating biosensor in each well of the 384-well assay plate intercepts broadband light and measures changes in resonant wavelengths (in picometers, pm) when a ligand binds to the target protein immobilized on the biosensor. To identify chemical binders of the $PGRN_{588-593}$ peptide, we immobilized the peptide onto the biosensor surface of Epic® assay plates using an amine-coupling method, and screened 4,800 compounds. For immobilization, 150 μg/ml peptide diluted in a 20 mM sodium acetate buffer, pH 5, was added to the assay plate to incubate for overnight at 4 °C. The plate was then washed in binding buffer (PBS with 5% DMSO) for three times and equilibrated in the binding buffer for 6 h in the Epic® carousal. During the equilibration period, compounds from the library stock plate (CNS-set compound library from Chembridge®) were diluted to 50 μM in binding buffer. Each compound was tested in quadruplicate. After the equilibration, a Baseline Reading was acquired by the Epic® spectrophotometer. Then equal volume of compounds was added to the equilibrated assay plate to a final concentration of 25 μM. After mixing and a 30 min 'binding' period, a Final Reading was acquired. The Specific Binding Signal (SBS) for the binding buffer-only control and individual compounds were calculated by subtracting the Final Reading to the Baseline Reading. Compounds that had a $PGRN_{588-593}$ binding response higher than the cutoff (i.e. mean of control + 3 standard deviations) were subjected to 2nd round dose-response assay for validation and determination of dissociation constant (Kd).

Molecular modeling of SORT1 with ligand binding

The X-ray structure for SORT1 (PDB code: 3F6K) was imported into the Protein-Preparation-Wizard GUI of Schrödinger with Maestro 2012 version 9.3.5 (Schrödinger, LLC) for adaption to the OPLS2005 force field. Bond orders were assigned, zero-order bonds to metals were determined, disulfide bonds were created as needed, and all hydrogens were re-generated for every residue. Hydrogen-bond assignment was based on sampling water orientations and taking into account crystallographic waters. Protonation states were predicted for pH 7.2 (range +/- 2.0) using PROPKA(1, 2). Steric clashes were resolved with convergence of RMSD to 0.3Å using SUthe OPLS2005 force field within Schrödinger-2013.

For modeling and molecular docking of peptide substrate based sequences (NTS, human $PGRN_{588\text{-}593}$, mouse $Pgrn_{584\text{-}589}$), starting conformations of substrates were obtained by Polak-Ribiere Conjugate Gradient (PRCG) energy minimization(3) with the OPLS 2005 force field for 5000 steps, or until the energy difference between subsequent structures was <0.001 kJ/mol-Ǻ (3, 8). Force field minimization used a water-based solvent, generating charges with an extended cutoff (VdW 8.0 Å, electrostatic 20 Å, H-bond 4.0 Å). We placed soft restraints on all residues >8 Å from the modeled substrate by using harmonic restraints at 100 kcal/mol, and allowed the residues within the 8 Å cutoff to move freely during serial PRCG energy minimization over 500 iterations with repetition as necessary to converge upon a gradient threshold of <0.05.

Grid of SORT1 with neurotensin ELYENKPRRPYIL and fragments --RRPYIL, –PYIL, or –YIL— A grid was generated for the docking site based upon the X-ray structure for SORT1 (all within grid for 3F6K; forming 216 sites) with neurotensin (NTS) ELYENKPRRPYIL and fragments --RRPYIL, –PYIL, or –YIL including these key residues from 3F6K: Lys227, Tyr271, Ser272, Phe273, Gly274, Leu275, Phe281, Ser283, Arg292, Ile294, Phe317, Tyr318, Ser319,

Ile320, Leu321, Tyr362, Thr369, Met330. Critical residues for docking and structural inhibition, which include residues Ser283-OH, Arg292-H_2N^+, 2x(Tyr318-NH), and Tyr318=O at the carboxylate and amino of the C-terminal Leucine (NTS) and Lys227-NH_3^+ with the Tyrosine carbonyl (NTS), were used to form the GRID of (nx, ny, nz) = (>100, >100, >100) Å.

Grid of SORT1 with human $PGRN_{588\text{-}593}$ –ALRQLL or mouse $PGRN_{584\text{-}589}$ –VPRPLL—A grid was generated for the docking site based upon the X-ray structure for SORT1 (all within grid for 3F6K; forming 216 sites) using superposition algorithm at the terminal leucine residues from Human or Mouse sequence, which then served as a Grid for subsequent docking, consistent with the protocol used for the NTS peptides.

Docking protocol – We have previously described the methodology used for substrate docking(4); briefly, the binding site was generated via overlapping grids based on the X-ray structure with a default rectangular box centered on the target substrate. Substrate peptides were docked into the binding site of SORT1 using Glide extra precision (SP, XP) (Glide, version 5.6, Schrödinger, LLC); molecular conformations were sampled using methods we have described previously(5). A structure-based pharmacophore score was generated from the optimized, best scoring pose for each substrate peptide based on the descriptors from Glide XP score using established approaches(4-6). The energetic value assigned to each pharmacophore feature was calculated using Phase (Phase, v3.2, Schrödinger, LLC) as the sum of the Glide XP contributions of the atoms comprising the site (SiteMap). Overall dockings at the active site were quantified and ranked on the basis of these energetic terms(6, 7). To account for protein flexibility and lessen the effects of minor steric clashes, excluded volumes spheres corresponding to 80% of the VdW atomic radii were created for all SORT1 atoms within 6 Å of each peptide substrate. A

minimum of ten poses per peptide with multiple input conformations generated, chosen for a combination of best-scoring features, was selected for visual and energetic comparison(4, 6, 7). Additionally, docking was enhanced with hydrophobic mapping, flexible docking, and Epik state penalities with OPLS2005 force field. The top poses were compared with results from the InducedFit method of Schrodinger, which accounts for enzyme flexibility upon binding. The pKas for the enzyme were broken into 224 overlapping clusters including waters from the X-ray structure, which allowed sampling of all possible combinations of pKa of 3F6K, was predicted for the given pH range and based on overall lowest energy from minimization of the hydrogens for SORT1(7-9).

Decomposition of individual interactions between SORT1 and NTS, human $PGRN_{588\text{-}593}$**, mouse** $Pgrn_{584\text{-}589}$ **peptides**

Using an average model for multiple docked poses of NTS, we find the following interactions to be informative on the docking scores.

NTS and SORT1 – The terminal leucine carboxylate from NTS gives a result consistent with the X-ray structure from 3F6K, where the carboxylate anion and carbonyl oxygen, O- and C=O, respectively, form strong interactions with the guanidinium moiety from Arg292, hydroxyl of Ser283, and amide hydrogen of Phe317, which is further stabilized by the carbonyl oxygen from Tyr318 and NTS terminal amide donor hydrogen. NTS terminal leucine side chain has hydrophobic proximity with Il3294 side chain atoms CB, CG1, CG2, and CD1 and Phe273 side chain atoms CG, CD1, CE1, and CZ. Tyr11 has pronounced interaction to Leu275 nitrogen (backbone) and to some extent Lys227 Nz group (side chain). Arg10 guanidinium (side chain) and Leu321 (SORT1) show strong electrostatic interaction. The backbone of SORT1 residues

Lys7 to Arg9 participate in interactions with water atoms that help stabilize NTS to the face of SORT1 that consists of residues Phe317, His360, Tyr362, Thr369, Asp370, Phe371, Thr372, Asn373, and Thr375. NTS Arg9 (side chain) and Phe371 (carbonyl oxygen) interact via charge interaction, while Thr372, Asn373, and Thr375 form hydrophobic packing with Asn6 and Lys7 aliphalic atoms. Strongly bound waters are present at NTS carbonyl oxygen atoms from residues Asn6, Pro8, and Arg9.

Human $PGRN_{588\text{-}593}$ and SORT1 – The terminal leucine carboxylate from human $PGRN_{588\text{-}593}$ gives a result consistent with that observed for NTS. The terminal leucine side chain has hydrophobic proximity with Il3294 side chain atoms CB, CG1, CG2, and CD1 and Phe273 side chain atoms CG, CD1, CE1, and CZ. The penultimate leucine has similar hydrophobic proximal interaction with Phe317 atoms CD1, CG, CE1, and CD2. The antepenultimate residue Gln for $PGRN_{588\text{-}593}$ is positioned outware into the aqueous pocket, but the Gln amide nitrogen has H-bonding with Ser319 hydroxyl (-OH). The preantepenultimate Arg residue encounters strong interactions via its guanidinium side chain and Gly366 (carbonyl carbon) and Glu368 (side chain atom OE1). The $PGRN_{588}$ residue (-NH) has favorable interactions with Phe371 (carbonyl oxygen). To a lesser degree, various packing atoms between the aliphatic atoms from the $PGRN_{588\text{-}593}$ peptide and the SORT1 surface allow for increased stability from van der Waals and Lennard-Jones type interactions.

Mouse $Pgrn_{584\text{-}589}$ and SORT1 – The terminal residue for mouse $Pgrn_{584\text{-}589}$ has similar carboxylate interactions to that found in both NTS and human $PGRN_{588\text{-}593}$, however there is a loss of the amide nitrogen (terminal Leu) with Tyr318. The terminal leucine alpha carbon from

$Pgrn_{584\text{-}589}$ forms interaction pair with Ser272 (atom CB2). The terminal leucine side chain atom CG forms interaction pair with Ile294 (atom CD1) and Tyr318 (atom CB).

Due to the strong carbonyl oxygen from the penultimate leucine ($Pgrn_{584\text{-}589}$) with Ser272, the remainder of the $Pgrn_{584\text{-}589}$ chain (residues VPR) is pulled toward the other face of SORT1, which includes residues Ser272, Phe273, Gly274, Leu275, Phe281, Ala282, Ser283, Val284, Arg293, Ile294, and Thr291. The resultant docked position for msP6aa is a shift in the dihedral angles along the backbone atoms from Pro4 (N, CA, C)—Leu 5 (N) and Pro (CA, C)—Leu (N, CA), which leaves residues $Pgrn_{584\text{-}589}$ (Val1 to Arg3) without strong interactions to SORT1. In some docking poses, $Pgrn_{584\text{-}589}$ residues VPR do shift toward the other SORT1 face, which includes residues Val1 (side chain) to Thr364 (carbonyl oxygen), Pro2 (side chain) with Phe317 (ring atoms)/Gly (backbone), and Arg3 (guanidinium) with Glu368 (side chain). However, this causes a loss in stability at the terminal leucine carboxylate and thus can be discarded as insufficient for maintaining $Pgrn_{584\text{-}589}$ in the pocket.

SUPPLEMENTARY REFERENCES

1 Li, H., Robertson, A.D. and Jensen, J.H. (2005) Very fast empirical prediction and rationalization of protein pKa values. *Proteins: Structure, Function, and Bioinformatics*, **61**, 704-721.

2 Søndergaard, C.R., Olsson, M.H.M., Rostkowski, M. and Jensen, J.H. (2011) Improved Treatment of Ligands and Coupling Effects in Empirical Calculation and Rationalization of pKa Values. *Journal of Chemical Theory and Computation*, **7**, 2284-2295.

3 Polak, E. and Ribiere, G. (1969).

4 Caulfield, T. and Medina-Franco, J.L. (2011) Molecular dynamics simulations of human DNA methyltransferase 3B with selective inhibitor nanaomycin A. *Journal of structural biology*, **176**, 185-191.

5 Caulfield, T. and Devkota, B. (2012) Motion of transfer RNA from the A/T state into the A-site using docking and simulations. *Proteins*, **80**, 2489-2500.

6 Loving, K., Salam, N.K. and Sherman, W. (2009) Energetic analysis of fragment docking and application to structure-based pharmacophore hypothesis generation. *Journal of computer-aided molecular design*, **23**, 541-554.

7 Salam, N.K., Nuti, R. and Sherman, W. (2009) Novel method for generating structure-based pharmacophores using energetic analysis. *Journal of chemical information and modeling*, **49**, 2356-2368.

8 Li, Z., Caulfield, T., Qiu, Y., Copland, J.A. and Tun, H.W. (2012) Pharmacokinetics of bendamustine in the central nervous system: chemoinformatic screening followed by validation in a murine model. *MedChemComm*, **3**, 1526-1530.

9 Perola, E., Walters, W.P. and Charifson, P.S. (2004) A detailed comparison of current docking and scoring methods on systems of pharmaceutical relevance. *Proteins*, **56**, 235-249.
